# Long-term hospitalized elderly people have lowered L-carnitine and coenzyme Q_10_ in blood than healthy elderly people

**DOI:** 10.1186/2049-3002-2-S1-P72

**Published:** 2014-05-28

**Authors:** Michiyo Takahashi, Toshikazu Suzuki, Hikaru Matsumoto, Hitoshi Nukada, Naotaka Hashizume

**Affiliations:** 1 Graduate School of Human Ecology, Wayo Women’s University, Ichikawa, Chiba, Japan; 2 Department of Health and Nutrition, Wayo Women’s University, Ichikawa, Chiba, Japan; 3 The Nukada Institute for Medical and Biological Research, Chiba, Japan; 4 Department of Health and Nutrition, University of Human Arts and Sciences, Saitama, Japan

## Background

Non-essential nutrients, L-carnitine (carnitine) and coenzyme Q_10_ (CoQ_10_) have attracted much attention as supplements for anti-aging and improvement of metabolism. Although the levels of carnitine and CoQ_10_ in the body decrease with advancing age, dietitians do not consider intakes of these nutrients when they plan food prescription or menu for elderly people. In addition, there are few reports comparing nutritional status of these nutrients between hospitalized and independent elderly people. In this study, the blood levels of carnitine and CoQ_10_ of long-term hospitalized and independent elderly people were investigated.

## Materials and methods

The participants were 15 elderly adults (4 men and 11 women, mean age 76.5±6.2 years) who were hospitalized for a long time, and 30 healthy elderly adults (9 men and 21 women, mean age 77.6±6.5 years). Inpatients were further divided into two groups (inpatients oral-feeding and inpatients enteral-feeding). Anthropometric parameters, nutrition intakes and biochemical blood tests of the participants were determined.

## Results

The level of serum albumin in inpatients oral-feeding, inpatients enteral-feeding, and healthy elderly adults were 3.4±0.3 g/dL, 3.4±0.4 g/dL, and 4.5±0.3 g/dL, respectively. The blood levels of carnitine in inpatients oral- and enteral-feeding were decreased by 25% (p <0.001) and 50% (p <0.001), respectively, as compared to that in independent elderly people. The blood levels of CoQ_10_ in inpatients oral- and enteral-feeding were decreased by 40% (p <0.01) and 50% (p <0.001), as compared to that in independent elderly people.

## Conclusion

The blood levels of carnitine and CoQ_10_ in the long-term hospitalized elderly people were lower than that in the healthy elderly people. Their blood levels in inpatients enteral-feeding were the lowest among 3 groups. The results suggest that carnitine and CoQ_10_ might decrease in both biosynthesis and intakes in long-term hospitalized elderly people. Therefore, supplementation of these non-essential nutrients to hospitalized elderly people might be effective for prevention of deterioration in their nutritional status.

